# Temperature and nutrition do not interact to shape the evolution of metabolic rate

**DOI:** 10.1098/rstb.2022.0484

**Published:** 2024-02-26

**Authors:** Lesley A. Alton, Teresa Kutz, Candice L. Bywater, Emily Lombardi, Fiona E. Cockerell, Sean Layh, Hugh Winwood-Smith, Pieter A. Arnold, Julian E. Beaman, Greg M. Walter, Keyne Monro, Christen K. Mirth, Carla M. Sgrò, Craig R. White

**Affiliations:** ^1^ Centre for Geometric Biology, Monash University, Melbourne, Victoria 3800, Australia; ^2^ School of Biological Sciences, Monash University, Melbourne, Victoria 3800, Australia

**Keywords:** life history, metabolic cold adaptation, experimental evolution, Krogh's rule, sex-specific effects

## Abstract

Metabolic cold adaptation, or Krogh's rule, is the controversial hypothesis that predicts a monotonically negative relationship between metabolic rate and environmental temperature for ectotherms living along thermal clines measured at a common temperature. Macrophysiological patterns consistent with Krogh's rule are not always evident in nature, and experimentally evolved responses to temperature have failed to replicate such patterns. Hence, temperature may not be the sole driver of observed variation in metabolic rate. We tested the hypothesis that temperature, as a driver of energy demand, interacts with nutrition, a driver of energy supply, to shape the evolution of metabolic rate to produce a pattern resembling Krogh's rule. To do this, we evolved replicate lines of *Drosophila melanogaster* at 18, 25 or 28°C on control, low-calorie or low-protein diets. Contrary to our prediction, we observed no effect of nutrition, alone or interacting with temperature, on adult female and male metabolic rates. Moreover, support for Krogh's rule was only in females at lower temperatures. We, therefore, hypothesize that observed variation in metabolic rate along environmental clines arises from the metabolic consequences of environment-specific life-history optimization, rather than because of the direct effect of temperature on metabolic rate.

This article is part of the theme issue ‘The evolutionary significance of variation in metabolic rates’.

## 1. Introduction

The effect of environmental temperature on ectotherm metabolic rates has been studied for over a century, beginning with the work of Ege & Krogh [1], who observed that the metabolic rate of a goldfish increased approximately exponentially with an acute increase in temperature. This relationship between metabolic rate and temperature has since been demonstrated in many ectothermic taxa, with metabolic rate typically increasing by a factor of 2–3 for every 10°C increase in temperature (known as the *Q*_10_ value) [2–4]. While observing this strong acute effect of temperature on the metabolic rate of a goldfish, Krogh also noted that the goldfish became very sluggish at low temperatures. This led Krogh to speculate that at low temperatures, fish from polar environments should exhibit relatively high metabolic rates compared with fish from temperate or tropical environments because, unlike the goldfish, polar fish remain active at very low temperatures [2,5].

In the 110 years since Ege & Krogh [1] measured their goldfish, Krogh's hypothesis has become known as metabolic cold adaptation [6] or Krogh's rule [7], and has generated significant controversy. Krogh's rule predicts that the metabolic rate of a population or species measured at any given common temperature should be monotonically negatively related to the temperature at which the population or species lives. However, macrophysiological studies comparing the metabolic rate of species or populations living along latitudinal clines offer mixed support for Krogh's rule [8–18].

When the macrophysiological pattern described by Krogh's rule is observed, it is regarded as an example of countergradient variation where phenotypic similarity along an environmental gradient arises as a consequence of genetic influences opposing environmental influences [19]. In the specific case of Krogh's rule, it is hypothesized that natural selection counteracts the acute effect of temperature on metabolic rate by favouring genotypes with relatively high metabolic rates at low temperatures and genotypes with relatively low metabolic rates at high temperatures.

By contrast to the expectations of Krogh's rule, Clarke [20–22] argued on philosophical grounds that there is no *a priori* reason to expect selection to favour relatively high metabolic rates at low temperatures because there is no benefit to increasing ATP production (and thereby oxygen consumption, a common indirect proxy of metabolic rate [23]) for the sake of it. Instead, Clarke expected that animals should adjust the rates of physiological processes that use ATP (e.g. ion pump activity, muscular and neural activity, growth and reproduction, waste excretion and locomotor activity involved in predator escape, mate and food acquisition) to suit a particular environmental context. What emerges from this argument is the expectation that selection should favour the rates of ATP production and utilization that maximize Darwinian fitness in a given environment. This premise is supported by recent work demonstrating that the relationship between metabolic rate and fitness traits is context dependent (e.g. [24–30]), and that variation in metabolic rate is linked to variation in growth and reproduction [31]. Thus, the abiotic and biotic variables that change along clines, including temperature, might give rise to clines in physiological traits, but these physiological clines do not arise as a direct effect of temperature itself as expected under Krogh's rule *senso stricto*.

Several attempts to test Krogh's rule have been undertaken, and the differences between evolutionary responses to temperature in the field and laboratory are informative. In the field, threespine stickleback (*Gasterosteus aculeatus*) and freshwater invertebrates that live in geothermally warmed systems exhibit reduced metabolic rates [32,33]. These findings offer support for Krogh's rule, but laboratory natural selection imposed by manipulation of temperature alone produces no evolved changes in metabolic rate in *Drosophila melanogaster* [9,34] and medaka fish, *Oryzias latipes* [35], and increased metabolic rates in warm environments in *Drosophila simulans* [36]. Taken together, these findings suggest that the correlation between metabolic rate and temperature along latitudinal clines [8,12–14,16] and in geothermally warmed systems [32,33] does not arise as a direct consequence of temperature alone, but rather as a consequence of the combination of environmental factors that covary with temperature. Exactly which environmental factors are involved remains unclear, and so our understanding of the ultimate drivers of metabolic rate evolution remains incomplete.

Here, we advance on previous manipulative tests of Krogh's rule by examining how temperature interacts with the availability and nutritional quality of food to shape the evolution of metabolic rate in *D. melanogaster*. Given that the energy balance of animals depends on both energy demand and supply, it seems plausible that temperature, as a driver of energy demand, will interact with environmental determinants of energy supply to shape the evolution of metabolic rate. Metabolic rate is hypothesized to evolve in response to variation in the availability and quality of food [37–39]. For example, environments with low food availability are expected to favour genotypes with relatively low metabolic rates because they are more resistant to starvation owing to their lower maintenance costs [37]. By contrast, environments with high food availability are expected to favour genotypes with relatively high metabolic rates because they can maximize energy assimilation for growth and reproduction by having more metabolic machinery [37].

As with Krogh's rule, tests of the predicted relationships between metabolic rate and food quality and quantity yield conflicting results. There is a positive relationship between net primary productivity (NPP), a determinant of food availability, and metabolic rate in *Peromyscus* mice [38], but no relationship between NPP and metabolic rate in birds [40]. Carnivorans with a higher proportion of vegetable matter in their diets have lower metabolic rates [41], but artificial selection for the ability to maintain body mass on a low-quality herbivorous diet results in no change in metabolic rate in bank voles [42]. Selection for increased starvation resistance in *Drosophila* results in higher body mass owing to increased lipid and carbohydrate storage and consequently a lower mass-specific metabolic rate [43,44]. However, under starved conditions, the metabolic rate of starvation-resistant flies is generally higher than that of control flies [45]. Taken together, these findings suggest that the evolutionary responses of metabolic rate to nutrition, like temperature, are complex, and likely context dependent.

The lack of any clear consensus on the evolutionary response of metabolic rate to either temperature or nutrition in isolation suggests that an experimental manipulation of these two factors simultaneously may be informative. Here, we evolved replicate lines of *D. melanogaster* for at least 24 generations in nine developmental environments representing a factorial combination of three temperatures (18, 25 and 28°C) and three diets (control, low-calorie and low-protein). The temperatures of 18 and 25°C broadly reflect the current seasonal temperature range (winter to summer) in the middle of the eastern Australian latitudinal cline where our *Drosophila* originated, and 28°C is representative of a 3°C future climate-warming scenario at this location [46]. The low-calorie and low-protein diets simulate reduced food abundance and nutritional quality, respectively, which are two forms of nutritional stress that animals are predicted to encounter because of human-induced environmental change, including that associated with climate change [47,48]. We imposed selection on pre-adult life stages only because, unlike highly mobile adults, pre-adult life stages are more restricted in their ability to select favourable conditions. In addition, by not imposing selection on adults and maintaining them under common garden conditions at 25°C on the control diet, we maximized the probability of population persistence for the duration of the experiment. This was necessary because temperature and nutrition interact to affect fecundity and viability [49–51].

After nearly 2 years, we examined the effect of our nine developmental selective environments on the evolution of metabolic rate by phenotyping 900 flies following two generations under common garden conditions at 25°C on the control diet. Phenotyping involved the measurement of metabolic rate (hereafter absolute metabolic rate) as the rate of carbon dioxide production at 25°C using flow-through respirometry. To disentangle the underlying mechanisms driving observed changes in absolute metabolic rate, we conducted simultaneous measures of activity and mass and accounted for the variance associated with these traits to estimate the mass-independent metabolic rates of inactive flies (hereafter, resting metabolic rate) as a measure of their minimum energy costs of self-maintenance. We phenotyped adult flies because thermal and nutritional conditions in the developmental environment affect the metabolic phenotype of adult flies [52], and because the metabolic phenotype of larval flies persists into adulthood [53].

Given that patterns resembling Krogh's rule are evident in nature for terrestrial insects [8], and for *D. melanogaster* specifically [9], but manipulations of temperature alone have failed to produce evolved differences in metabolic rate in *D. melanogaster* [9,34], we predicted that temperature and nutrition would interact to a produce a pattern resembling Krogh's rule. Specifically, we predicted that warm environments with poor nutrition (low-calorie and low-protein diets) would favour genotypes with relatively low metabolic rates to cope with the relatively high energy demand and poor energy supply. By contrast, cool environments with good nutrition (control diet) would favour genotypes with relatively high metabolic rates to maximize the benefits of the relatively low energy demand and good energy supply. We, therefore, predicted that when we compared animals from these extreme environments at a common temperature, a pattern resembling Krogh's rule might emerge.

## 2. Methods

### (a) Fly stock

Field-inseminated females of *D. melanogaster* were collected in January 2018 from Duranbah, Australia (28.3°S, 153.5°E), which is mid-way along the east coast of Australia. Two hundred of these females were isolated in separate culture vials to establish 200 independent isofemales lines (full-sib families). The second generation of each isofemale line was treated with tetracycline to remove *Wolbachia*. Five virgin females and males from the fourth generation of each isofemale line were pooled together to form the base population. The base population was maintained at 25°C on a 12 : 12 h light : dark cycle on the control diet (see below) and was expanded for two generations, resulting in 60 bottles each containing approximately 750–1000 flies.

### (b) Selective environments

**Figure 1.**
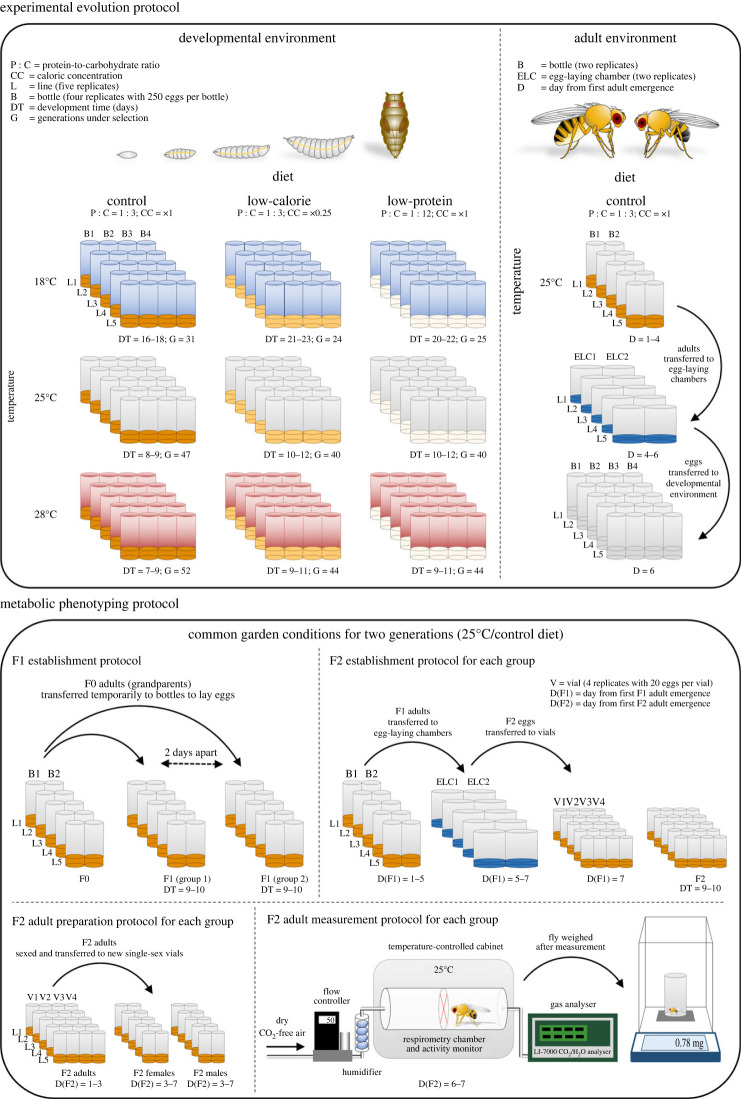
A schematic diagram of the experimental design.

From the base population, eggs were collected and divided among nine selective environments with five replicate lines per treatment. The nine selective environments were a full-factorial combination of three temperatures (18, 25 and 28°C) and three diets (control, low-calorie and low-protein) (figure 1).

Our lower experimental temperatures were chosen based on recent climate data (1970–2017) recorded at the location nearest where our *Drosophila* originated (Brisbane: 27.4°S, 153.1°E) (long-term station data downloaded from the Bureau of Meteorology, bom.gov.au). At this location, daily mean temperature ranges from 15°C (winter average: June–August) to 25°C (summer average: December­–February). Our highest experimental temperature was chosen to represent an end-of-century intermediate climate-warming scenario when global surface temperatures are estimated to be 2.7°C warmer [46].

Experimental diets were created by varying the quantities of inactive yeast (containing 45% protein, 33% carbohydrate and 1% fat), dextrose and potato flakes (containing 10% protein and 80% carbohydrate) added to 1.1 l of water. The control diet (40 g yeast, 30 g dextrose and 20 g potato) had a protein-to-carbohydrate ratio (P : C) of 1 : 3 and a caloric content of 1360 kJ. The low-calorie diet (10 g yeast, 7.5 g dextrose and 5 g potato) had the same P : C as the control diet but 25% of the calories (340 kJ). The low-protein diet (9 g yeast, 55 g dextrose and 20 g potato) had the same caloric content as the control diet, but 25% of the protein (a P : C of 1 : 12). Added to all diets were 7 g of agar and preservatives (12 ml of nipagen and 5 ml of propionic acid). The specific caloric concentrations and macronutrient ratios of our diets were chosen based on our previous study showing that larval survival is reduced under these conditions [51].

### (c) Experimental evolution protocol

At the beginning of each generation, each line was established with 1000 eggs divided among four 300 ml bottles each containing 250 eggs and 62.5 ml of the experimental diet. Bottles were maintained at the experimental temperature until all adults emerged. Upon first emergence, all adults were collected daily over 2–3 days until no more adults emerged, to avoid selection for fast development. Adults were collected into two bottles and maintained at 25°C on the control diet. On the third day, adults were tipped into new bottles with fresh medium. In the afternoon of the fourth day, adults were transferred to 250 ml egg-laying chambers containing approximately 11 ml of medium that was coloured with blue food dye and modified to prevent flies from burying their eggs (40 g yeast, 30 g dextrose and 10 g potato, 14 g agar, 12 ml nipagen, 5 ml propionic acid dissolved in 1.1 l of water), making eggs easily accessible for collection. The medium in the egg-laying chambers was coated in autoclaved yeast dissolved in water to encourage egg-laying behaviour. To ensure that adults laid enough eggs to establish the next generation, adults were acclimated to these egg-laying chambers for approximately 24 h, with fresh medium provided on the morning of the fifth day. After the 24 h acclimation period, adults were provided with fresh medium and allowed to lay eggs overnight. The eggs collected on the morning of the sixth day were used to establish the next generation. Owing to differences in development time associated with temperature and diet (7–23 days), the number of generations over which selection occurred varied from 24 to 52 among our selective environments (electronic supplementary material, table S1). A schematic diagram of the experimental evolution protocol is provided in figure 1.

### (d) Metabolic phenotyping

To assess the effects of developmental temperature and diet on the evolution of metabolic rate in adult flies, all lines were maintained under common garden conditions at 25°C on the control diet for two generations prior to metabolic phenotyping. These common garden conditions were chosen because they match the standard laboratory rearing conditions for *D. melanogaster*, which have been selected because flies reproduce and survive well under these conditions [49–51]. Logistical constraints prevented us from maintaining and measuring flies under other common garden conditions. To generate flies of the same age for metabolic phenotyping, the grandparents from each selective environment continued to be maintained in bottles until the grandparents from all selective environments had been collected. Grandparents of varying ages were then used to produce two groups of parents of the same age by allowing grandparents to oviposit in bottles on one day (to produce the first parent group), and then again in new bottles 2 days later (to produce the second parent group) (electronic supplementary material, table S1). Grandparents oviposited in bottles until approximately 250 eggs were visible. The parents that emerged from these bottles were collected, maintained in separate groups, and used to produce a second generation following the protocols for experimental evolution with the exception that adults were transferred to egg-laying chambers a day later. The second generation of each line was established with 80 eggs divided among four vials each containing 20 eggs and 6.8 ml of medium. Upon emergence, adults were collected into four vials over 2 days. On the third day, adults were sexed under CO_2_ anaesthesia, after which females and males were maintained separately in four vials (two vials per sex) until measurements began on the sixth day post first emergence in February 2020. A schematic diagram of the protocol for establishing experimental flies for metabolic phenotyping is provided in figure 1.

The rates of CO_2_ production (${\dot{V}}_{{\mathrm{CO}}_{2}}$, μl h^−1^) of individual male and female flies at 25°C were measured as a proxy for metabolic rate using a 16-channel flow-through respirometry (indirect calorimetry) system described by Alton *et al.* [52] and Alton & Kellermann [54] (see electronic supplementary material for details). The activity of individual flies was measured simultaneously using *Drosophila* activity monitors (DAM) that counted the number of times a fly broke an infrared beam when it walked past the midpoint of the respirometry chamber, which was a plastic tube with a 5 mm diameter and 45 mm of tube length available for voluntary walking locomotion. The ${\dot{V}}_{{\mathrm{CO}}_{2}}$ and activity of 16 flies were measured in one measurement block with 16 respirometry chambers divided evenly between two DAMs. Both DAMs were placed inside a temperature-controlled cabinet that maintained temperature to 25 ± 1°C and kept flies in the dark. The ${\dot{V}}_{{\mathrm{CO}}_{2}}$ and activity of each fly were measured continuously for 30 min following a 50 min settling period inside the chamber without food. The lowest ${\dot{V}}_{{\mathrm{CO}}_{2}}$ averaged over 10 min during this 30 min measurement period was taken as the measure of absolute metabolic rate. The activity data recorded during the same 10 min period that was selected for the absolute metabolic rate calculation was taken as the measure of activity for the fly, which equated to the number of times the fly walked past the midpoint of the chamber per minute (activity rate, beam breaks min^−1^). Visualization of the relationship between ${\dot{V}}_{{\mathrm{CO}}_{2}}$ and activity rate indicated that, while most flies were active during measurements, a small number of flies were relatively inactive and had low ${\dot{V}}_{{\mathrm{CO}}_{2}}$ values. We, therefore, chose to exclude flies with activity rates less than 0.75 beam breaks min^−1^ (5 males and 14 females) as these flies were in a metabolic state that was different from most flies.

Immediately following metabolic rate measurements, the wet mass of flies was recorded to the nearest 0.01 mg (XS105DU Analytical Balance, Mettler Toledo, Greifensee, Switzerland). Flies were then frozen at –20°C and later dried at 60°C for 40 h. Immediately after drying, the dry mass of flies was recorded to the nearest 0.001 mg (XP2U Ultra Micro Balance, Mettler Toledo, Greifensee, Switzerland). The body water fraction of flies was calculated by subtracting their dry mass from their wet mass and dividing by their wet mass.

Metabolic rate measurements were conducted blind to treatment groups over four consecutive days, with 224–226 flies measured across 14–15 measurement blocks each day (a total of 900 flies). The first 2 days were used to measure the adult progeny from the first parent group, and the final 2 days were used to measure the adult progeny from the second parent group. One female and one male from each line were measured in a randomized order over the first six measurement blocks. This was repeated another four times over the 2 days so that a total of five females and five males from each line were measured from each parent group. Flies were 4–6 and 5–7 days of age on the first and second day of measurement, respectively.

### (e) Statistical analyses

All data were analysed using R v.4.2.3 [55]. The interactive effects of temperature and diet on the evolution of adult traits were analysed separately for each sex because there is no overlap in the mass range of females (1.18–2.09 mg) and males (0.60–1.07 mg) (i.e. mass and sex are collinear and perfectly confounded) [56]. Linear mixed models were fitted to log_10_-transformed mass and log_10_-transformed absolute metabolic rate data using the *lmer* function of the *lme4* package v.1.1-33 [57]. Generalized linear mixed models were fitted to activity count data and body water fraction data using the *glmmTMB* function of the *glmmTMB* package v.1.1.7 [58]. For activity, the model used a negative binominal (linear parameterization) distribution and the natural log of measurement duration was used as an offset variable. For body water fraction, the model used a beta distribution and logit link function. Models used sum-to-zero contrasts and restricted maximum likelihood for parameter estimation.

Each model included the fixed factors of temperature (18, 25 or 28°C), diet (control, low-calorie or low-protein) and temperature–diet interaction, and random intercepts for lines (1–45), measurement channels (1–16) and measurement blocks (1–59). Absolute metabolic rate data were also analysed with mean-centred log_10_-transformed mass and mean-centred activity rate as continuous covariates to determine treatment effects on resting metabolic rate.

The significance of fixed effects was tested using Type-III *F*-tests with Kenward–Roger degrees of freedom for linear mixed models, and Type-III Wald *χ*^2^ tests for generalized linear mixed models, using the *Anova* function of the *car* package v.3.1-2 [59]. The *emmeans* package v.1.8.5 [60] was used to calculate estimated marginal means and to perform *post hoc* comparisons of means with Kenward–Roger degrees of freedom and *p-*values adjusted for multiple testing using Tukey's method.

## 3. Results

**Figure 2.**
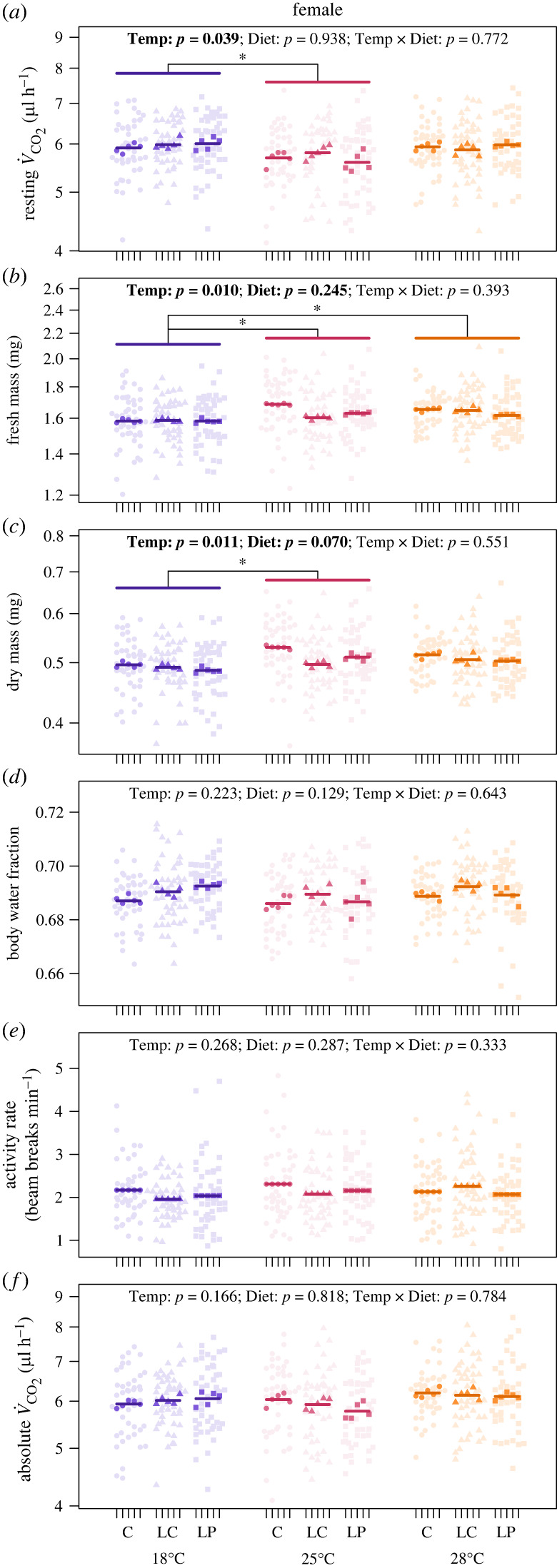
The effect of developmental temperature (Temp: 18, 25 or 28°C) and diet (control: C; low-calorie: LC or low-protein: LP) on the evolution of adult traits in female *Drosophila melanogaster*. Adult traits are resting metabolic rate (rate of CO_2_ production, ${\dot{V}}_{{\mathrm{CO}}_{2}}$, μl h^−1^) (*a*), fresh mass (*b*), dry mass (*c*), body water fraction (*d*), activity rate (beam breaks min^−1^) (*e*) and absolute metabolic rate (*f*). Light points are individual measurements, dark points are line means and horizontal bars are treatment means (see Methods for details). Data points for resting ${\dot{V}}_{{\mathrm{CO}}_{2}}$ are the measured ${\dot{V}}_{{\mathrm{CO}}_{2}}$ values standardized to the mean log_10_-transformed fresh mass (mean fresh mass = 1.62 mg) and zero activity levels based on model parameter estimates for log_10_-transformed mass and activity rate (electronic supplementary material, table S2). The statistical significance of main fixed effects (Temp and Diet) and the temperature–diet interaction (Temp × Diet) is indicated by *p-*values, with those less than 0.05 highlighted in bold. Asterisks indicate statistically significant differences between temperature treatments as determined by *post hoc* analyses.

We found no statistically significant interaction between developmental temperature and diet on the evolution of any of the traits measured in adult females or males (electronic supplementary material, tables S2 and S3). However, there was a significant effect of temperature on the evolution of the resting metabolic rate and body mass of adult females (electronic supplementary material, table S2). The resting metabolic rate of females evolved at 18°C was 5% higher than that of those evolved at 25°C (*t*_37.71_ = 2.46, *p* = 0.048), but similar to that of those evolved at 28°C (*t*_35.80_ = 0.36, *p* = 0.931), and the resting metabolic rate of females evolved at 25 and 28°C was similar (*t*_36.15_ = –2.13, *p* = 0.097) (figure 2*a*). The fresh mass of females evolved at 18°C was 3% lower than that of those evolved at 25°C (*t*_36.29_ = –2.78, *p* = 0.023) and 28°C (*t*_35.04_ = –2.83, *p* = 0.020), and the fresh mass of females evolved at 25 and 28°C was similar (*t*_36.19_ = –0.02, *p* = 1.000) (figure 2*b*). The dry mass of females evolved at 18°C was 4% lower than that of those evolved at 25°C (*t*_36.44_ = –3.01, *p* = 0.013), but similar to that of those evolved at 28°C (*t*_35.44_ = –2.44, *p* = 0.051), and the dry mass of females evolved at 25 and 28°C was similar (*t*_36.17_ = 0.59, *p* = 0.825) (figure 2*c*). There was no significant effect of temperature or diet on the evolution of the body water fraction (figure 2*d*), activity (figure 2*e*) or absolute metabolic rate (figure 2*f*) of females (electronic supplementary material, table S2).

**Figure 3.**
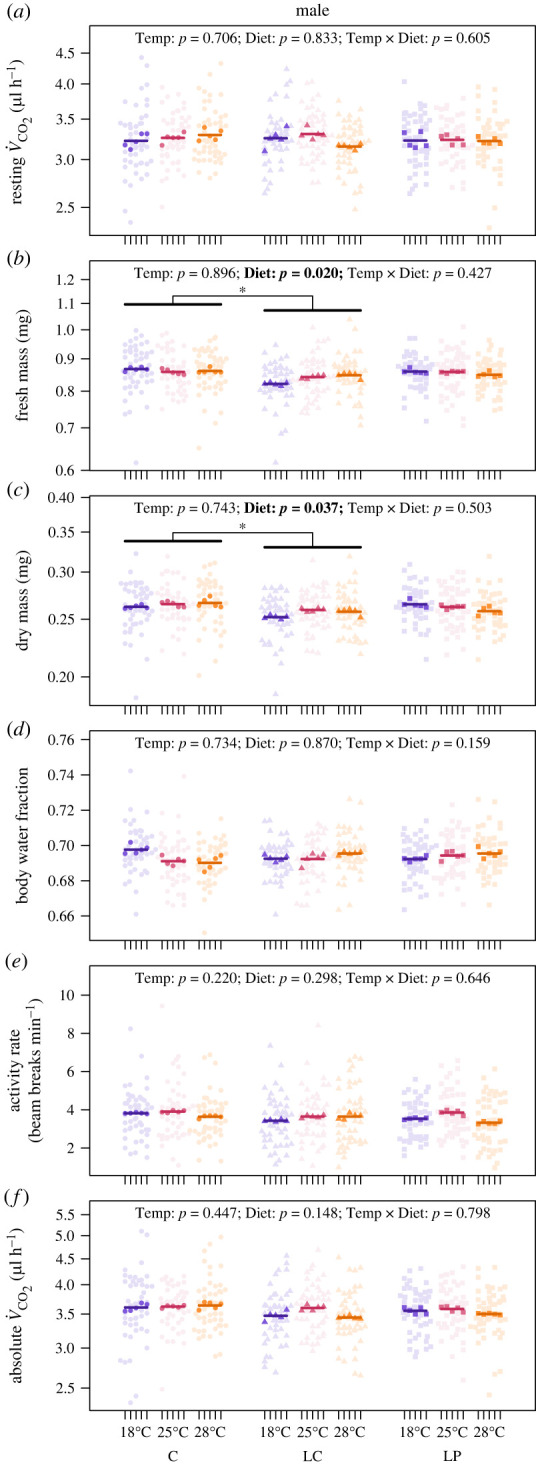
The effect of developmental temperature (Temp: 18, 25 or 28°C) and diet (control: C; low-calorie: LC or low-protein: LP) on the evolution of adult traits in male *Drosophila melanogaster*. Adult traits are resting metabolic rate (rate of CO_2_ production, ${\dot{V}}_{{\mathrm{CO}}_{2}}$, μl h^−1^) (*a*), fresh mass (*b*), dry mass (*c*), body water fraction (*d*), activity rate (beam breaks min^−1^) (*e*) and absolute metabolic rate (*f*). Light points are individual measurements, dark points are line means and horizontal bars are treatment means (see Methods for details). Data points for resting ${\dot{V}}_{{\mathrm{CO}}_{2}}$ are the measured ${\dot{V}}_{{\mathrm{CO}}_{2}}$ values standardized to the mean log_10_-transformed fresh mass (mean fresh mass = 0.85 mg) and zero activity levels based on model parameter estimates for log_10_-transformed mass and activity rate (electronic supplementary material, table S3). The statistical significance of main fixed effects (Temp and Diet) and the temperature–diet interaction (Temp × Diet) are indicated by *p-*values, with those less than 0.05 highlighted in bold. Asterisks indicate statistically significant differences between diet treatments as determined by *post hoc* analyses.

In males, there was no effect of temperature or diet on the evolution of resting metabolic rate (figure 3*a*), but there was a significant effect of diet on the evolution of body mass (electronic supplementary material, table S3). The fresh mass of males evolved on the low-calorie diet was 3% lower than that of those evolved on the control diet (*t*_35.19_ = 2.86, *p* = 0.019), but similar to that of those evolved on the low-protein diet (*t*_35.43_ = –2.11, *p* = 0.103), and the fresh mass of males evolved on the control and low-protein diets was similar (*t*_36.31_ = 0.74, *p* = 0.740) (figure 3*b*). The dry mass of males evolved on the low-calorie diet was 3% lower than that of those evolved on the control diet (*t*_34.84_ = 2.65, *p* = 0.032), but similar to that of those evolved on the low-protein diet (*t*_35.21_ = –1.74, *p* = 0.203), and the dry mass of males evolved on the control and low-protein diets was similar (*t*_35.15_ = 0.89, *p* = 0.647) (figure 3*c*). There was no significant effect of temperature or diet on the evolution of the body water fraction (figure 3*d*), activity (figure 3*e*) or absolute metabolic rate (figure 3*f*) of males (electronic supplementary material, table S3).

## 4. Discussion

Environmental temperature is well known to have a strong influence on the metabolic rate of ectotherms, and Krogh's rule offers a framework not only to understand how temperature has shaped the historical evolution of metabolic rate, but also to predict the consequences of ongoing climate warming. Previous laboratory natural selection experiments in *Drosophila* and medaka fish, *O. latipes*, have failed to replicate the pattern predicted by Krogh's rule [9,34–36], suggesting that temperature alone does not give rise to the monotonically negative relationship between metabolic rate and environmental temperature sometimes observed in insects and fish [8,13,14]. However, experimental evolution studies may fail to replicate clines observed in nature because laboratory environments are too simple [61]. Free-living animals face multiple abiotic and biotic challenges simultaneously; thus trait evolution in nature is more likely to be driven by interactions among multiple environmental factors [61]. In particular, the provisioning of ad libitum food in laboratory studies has been identified as a potential shortcoming of studies attempting to understand the relationship between metabolic rate and fitness [24,37]. We, therefore, chose to explore the effects of temperature on the evolution of metabolic rate under a range of nutritional conditions to see if we could generate the pattern predicted by Krogh's rule.

Surprisingly, we found no evidence for an interactive effect of temperature and nutrition, nor a significant main effect of nutrition, on the metabolic rate of adult *D. melanogaster*. This lack of a response could be because we imposed selection on pre-adult life stages but measured metabolic rates in adults. However, even though we did not impose selection on adults, we did see some responses to selection in adults, which adds further evidence that developmental conditions carry over to affect the metabolic phenotype of adults [52,54]. The response to selection observed in the present study provides limited support for Krogh's rule, but only for the resting metabolic rate of females and only at lower temperatures, with females evolved at 18°C having 5% higher resting metabolic rates than those evolved at 25°C (figure 2*a*). Females evolved at 28°C had similar resting metabolic rates to those evolved at 18 and 25°C (figure 2*a*), suggesting that there may be a limit to the extent that evolution can oppose the thermodynamic effects of warming, with some indication that warming may instead favour higher metabolic rates, as shown in *D. simulans* [36]. Unlike females, males exhibited no change in resting metabolic rate in response to temperature (figure 3*a*), which is consistent with the findings of our previous study [34] and that of Berrigan & Partridge [9]. Although Berrigan & Partridge [9] found that male flies evolved at 18°C had 5–7% higher mass-specific metabolic rates compared with those evolved at 25°C (a comparable effect size to what we observed in females in the present study), this effect was statistically non-significant when they accounted for the non-independence of flies from replicate lines.

In addition to the limited effects we observed on resting metabolic rate, we found that evolution at 18°C reduced the body mass of females (but not males) by 3% compared with those evolved at 25 and 28°C (figure 2*b*). We also found that evolution on a low-calorie diet reduced the body mass of males (but not females) by 3% compared with those evolved on the control diet (figure 3*b*). Our results contrast with that of Bochdanovits & de Jong [62], who found that evolutionary responses of body mass to a low-calorie diet varied with selection temperature in male *D. melanogaster*. However, our finding that adaptation to a low-calorie diet results in smaller male flies is consistent with that of other studies, although these other studies also report the same response in female flies [63,64]. Our observation that females evolved at 18°C are smaller contrasts with the findings of Partridge *et al.* [65], who found that female and male *D. melanogaster* evolved at 16.5°C have a larger thorax length and wing area compared with those evolved at 25°C. However, other studies have shown that male and female wing size does not evolve in response to temperature [66,67]. Unlike the finding of Partridge *et al.* [65], our finding that cold environments result in smaller females is in the opposite direction of the temperature–size rule, a pattern in which ectotherms mature at a larger size when reared in cooler conditions [68–71]. Thus, our finding represents an evolutionary response that could generate countergradient variation in body size in the field [19,72].

Our observation of relatively higher resting metabolic rates in females evolved at 18°C compared with those evolved at 25°C could also represent countergradient variation in metabolic rate, albeit occurring only at lower temperatures. However, the extent to which the sex-specific changes in mass and metabolic rate that we observed in the present study will result in size and metabolic clines in nature may (or may not) be constrained by sexual conflict, which can arise when different phenotypic optima are favoured in males and females [73,74]. This is expected to constrain adaptation because intersexual genetic correlations are typically high, which reduces the capacity of each sex to reach their unique selective optima, though the ultimate strength of this constraint will depend on both the strength of selection and the stability of the environment [75–78].

Why the resting metabolic rate and mass of females evolved only in response to temperature and the mass of males evolved only in response to diet is unclear. While it may seem plausible that these sex-specific responses could be related to differences in reproductive investment, females and males differ in many aspects, including their size (e.g. female *D. melanogaster* are larger, figures 2*b* and 3*b*) and behaviour (e.g. male *D. melanogaster* are more active, figures 2*e* and 3*e*). Thus the sex-specific effects observed in the present study could be related to any of the multitude of differences between females and males (e.g. [79–82]). Future work should explore the sex-specific evolution of life-history traits (e.g. development time, age at maturity, reproductive investment, rates of senescence and lifespan) in response to variation in temperature and nutrition to understand why we observed sex-specific metabolic responses in the present study.

### (a) Why are we unable to replicate Krogh's rule using laboratory natural selection?

Although we observed an evolutionary response to low temperatures in female flies that is consistent with Krogh's rule, the effect was small, not monotonically negative across all selection temperatures and absent in males (figures 2*a* and 3*a*). Our results, therefore, add to the growing number of experimental evolution studies that fail to find convincing evidence that the metabolic rate of ectotherms evolves in response to environmental temperature in the direction predicted by Krogh's rule [9,34–36]. Our study also shows that Krogh's rule does not emerge as a consequence of interactions between temperature and nutrition (figures 2*a* and 3*a*), which seems a surprising result given that directional relationships between metabolic rate and fitness are expected when nutritional conditions vary [37].

However, when we consider the selection protocol used in the present study, perhaps it is not surprising that we did not observe interactive effects of temperature and nutrition on trait evolution. In the present study, fly populations evolved in discrete generations where the initial egg density was controlled and constant among environments. Populations maintained at low temperatures and on nutritionally poor diets were given as much time as they needed to develop and all adults that emerged were given the opportunity to contribute to the next generation in a discrete egg-laying window. By employing these protocols we limited the confounding effects of density and selection on development time, but also accommodated the direct physiological effects of temperature and nutrition on population size and generation time. As such, our protocol might have eliminated selection pressures that act to oppose these effects in nature. For example, selection might favour relatively rapid development (and therefore high metabolic rates [31,83–85]) in cold environments (e.g. [86]) to reduce otherwise long generation times. Alternatively, or in addition, selection might favour low metabolic rates at high temperatures to increase otherwise low population carrying capacities [87–90]. We, therefore, propose that selection yields clines in life-history strategy (e.g. [91]), which in turn leads to clines in metabolic rate that are consistent with Krogh's rule [22,92].

We suggest that the next phase of experimental evolution studies that seek to explain clines in metabolic rate in nature should consider two complementary approaches: (i) artificial selection to generate replicate lines of animals that differ in metabolic rate, and then assess their relative fitness across a range of environments that mimic the conditions along clines, and (ii) laboratory natural selection to explore how metabolic rate evolves in warm and cold environments while constraining generation time to be similar across environments (i.e. select for faster development time in cold environments and slower development in warmer environments) and allowing population size to vary naturally.

## 5. Conclusion

Understanding how global climate change and other human-induced environmental changes will affect the energy expenditure of animals is one of the most pressing challenges facing physiological ecologists [4,31,52,54,93]. Past work has estimated the increase in ectotherm metabolic rates associated with the acute effect of recent climate warming [93], while other work has predicted that phenotypic plasticity is likely to counter that increase [4]. But more recently it has become clear that plastic responses may not be as effective as previously thought, with developmental nutrition and species interactions modifying metabolic responses to warming [52,54]. What remains unclear, however, is the extent to which evolutionary adaptation may act to alter metabolic rates in the face of future climate warming.

The consensus emerging from laboratory natural selection experiments is that temperature alone does not consistently drive evolutionary responses in metabolic rate [9,34–36]. Thus, the metabolic consequences of climate warming cannot be understood through simple manipulations of temperature alone. We instead hypothesize that to understand the metabolic costs of climate warming it will be necessary to understand how climate warming will shift life histories, and how these shifts will result in correlated changes in metabolic rate.

## Data Availability

Data and code are provided in the electronic supplementary material [94].
